# HIV and Sexually Transmitted Infections Among Persons with Monkeypox — Eight U.S. Jurisdictions, May 17–July 22, 2022

**DOI:** 10.15585/mmwr.mm7136a1

**Published:** 2022-09-09

**Authors:** Kathryn G. Curran, Kristen Eberly, Olivia O. Russell, Robert E. Snyder, Elisabeth K. Phillips, Eric C. Tang, Philip J. Peters, Melissa A. Sanchez, Ling Hsu, Stephanie E. Cohen, Ekow K. Sey, Sherry Yin, Chelsea Foo, William Still, Anil Mangla, Brittani Saafir-Callaway, Lauren Barrineau-Vejjajiva, Cristina Meza, Elizabeth Burkhardt, Marguerite E. Smith, Patricia A. Murphy, Nora K. Kelly, Hillary Spencer, Irina Tabidze, Massimo Pacilli, Carol-Ann Swain, Kathleen Bogucki, Charlotte DelBarba, Deepa T. Rajulu, Andre Dailey, Jessica Ricaldi, Leandro A. Mena, Demetre Daskalakis, Laura H. Bachmann, John T. Brooks, Alexandra M. Oster, Monkeypox, HIV, and STI Team, Monkeypox, HIV, and STI Team, Michael Abassian, Meaghan Abrego, David Addo, Bridget J. Anderson, Connie Austin, Kailey Bradley, David Bui, Shua Chai, Eric Chapman, Joseph Clement, Catherine Comis, Phoebe Danza, Marisa Donnelly, Kerri Dorsey, Kate Drezner, Alicia Dunajcik, Areesh Fatmee, Amanda Feldpausch, Lauren Finn, Rebecca Fisher, Kameron Gadawski, Jasmine Gaillard, Varun Gandhi, Amy Garlin, Sarah Gillani, Jamilla Green, Megan Hill, Taylor Holly, Virginia Hu, Otto Ike, Anna Satcher Johnson, Kelly Johnson, Janna Kerins, David Kern, Bita Khoshhal, Akiko Kimura, Irma Kocer, Colin Korban, Chun-Mai Kuo, Rodriques Lambert, Issa Lee-Hall, Jessica Lorenzo-Luaces, Elise Mara, Amy Marutani, Karla Miletti, Wilson Miranda, Allison Morrow, Dawn Nims, Melissa Ongpin, Chisom Onyeuku, Jessica Pavlick, Eugene Pennisi, Neela Persad, Mary Pomeroy, Kathleen Poortinga, Dylan Atchley Procter, Marisa Ramos, Eli Rosenberg, Lori Saathoff-Huber, Nannie Song, Dan Stowell, Deanna Sykes, Amanda Terminello, Ebony Thomas, Chris Toomey, Brittany Wilbourn, Tanya Williams, Pascale Wortley

**Affiliations:** ^1^CDC Monkeypox Emergency Response Team; ^2^DLH Corporation, Atlanta, Georgia; ^3^California Department of Public Health; ^4^San Francisco Department of Public Health, San Francisco, California; ^5^Los Angeles County Department of Public Health, Los Angeles, California; ^6^District of Columbia Department of Health; ^7^Georgia Department of Public Health; ^8^Illinois Department of Public Health; ^9^Epidemic Intelligence Service, CDC; ^10^Chicago Department of Public Health, Chicago, Illinois; ^11^New York State Department of Health.; California Department of Public Health; New York State Department of Health; District of Columbia Department of Health; New York State Department of Health; Illinois Department of Public Health; Georgia Department of Public Health; California Department of Public Health; California Department of Public Health; California Department of Public Health; San Francisco Department of Public Health; Georgia Department of Public Health; Los Angeles County Department of Public Health; California Department of Public Health; District of Columbia Department of Health; District of Columbia Department of Health; CDC Monkeypox Emergency Response Team; District of Columbia Department of Health; Georgia Department of Public Health; Los Angeles County Department of Public Health; Los Angeles County Department of Public Health; Georgia Department of Public Health; CDC Monkeypox Emergency Response Team; New York State Department of Health; San Francisco Department of Public Health; District of Columbia Department of Health; CDC Monkeypox Emergency Response Team; Georgia Department of Public Health; Chicago Department of Public Health; Los Angeles County Department of Public Health; CDC Monkeypox Emergency Response Team; CDC Division of HIV Prevention; California Department of Public Health; Chicago Department of Public Health; Chicago Department of Public Health; Georgia Department of Public Health; California Department of Public Health; CDC Monkeypox Emergency Response Team; Chicago Department of Public Health; Los Angeles County Department of Public Health; Georgia Department of Public Health; CDC Monkeypox Emergency Response Team; Georgia Department of Public Health; San Francisco Department of Public Health; Los Angeles County Department of Public Health; District of Columbia Department of Health; New York State Department of Health; District of Columbia Department of Health; Illinois Department of Public Health; San Francisco Department of Public Health; CDC Monkeypox Emergency Response Team; Georgia Department of Public Health; Georgia Department of Public Health; CDC Monkeypox Emergency Response Team; CDC Monkeypox Emergency Response Team; Los Angeles County Department of Public Health; Georgia Department of Public Health; California Department of Public Health; New York State Department of Health; Illinois Department of Public Health; California Department of Public Health; CDC Monkeypox Emergency Response Team; California Department of Public Health; CDC Monkeypox Emergency Response Team; CDC Monkeypox Emergency Response Team; San Francisco Department of Public Health; District of Columbia Department of Health; CDC Monkeypox Emergency Response Team; Georgia Department of Public Health.

High prevalences of HIV and other sexually transmitted infections (STIs) have been reported in the current global monkeypox outbreak, which has affected primarily gay, bisexual, and other men who have sex with men (MSM) (*1*–*5*). In previous monkeypox outbreaks in Nigeria, concurrent HIV infection was associated with poor monkeypox clinical outcomes (*6*,*7*). Monkeypox, HIV, and STI surveillance data from eight U.S. jurisdictions* were matched and analyzed to examine HIV and STI diagnoses among persons with monkeypox and assess differences in monkeypox clinical features according to HIV infection status. Among 1,969 persons with monkeypox during May 17–July 22, 2022, HIV prevalence was 38%, and 41% had received a diagnosis of one or more other reportable STIs in the preceding year. Among persons with monkeypox and diagnosed HIV infection, 94% had received HIV care in the preceding year, and 82% had an HIV viral load of <200 copies/mL, indicating HIV viral suppression. Compared with persons without HIV infection, a higher proportion of persons with HIV infection were hospitalized (8% versus 3%). Persons with HIV infection or STIs are disproportionately represented among persons with monkeypox. It is important that public health officials leverage systems for delivering HIV and STI care and prevention to reduce monkeypox incidence in this population. Consideration should be given to prioritizing persons with HIV infection and STIs for vaccination against monkeypox. HIV and STI screening and other recommended preventive care should be routinely offered to persons evaluated for monkeypox, with linkage to HIV care or HIV preexposure prophylaxis (PrEP) as appropriate.

Eight health departments matched probable and confirmed cases of monkeypox^†^ diagnosed through July 22, 2022, and occurring among persons aged ≥18 years, to local HIV and STI surveillance data using individually established methods that included various personal identifiers (e.g., name, soundex,^§^ date of birth, address, and telephone number). Matched data were deidentified and securely transmitted to CDC for analysis.

Among persons with monkeypox, prevalence of diagnosed HIV infection, determined through local HIV surveillance matches,^¶^ was calculated. HIV surveillance data were used to assess receipt of HIV care,** HIV viral suppression (an indication of antiretroviral therapy use),^††^ most recent CD4 count,^§§^**** and time since HIV diagnosis (*8*). STI surveillance data were used to assess chlamydia, gonorrhea, and syphilis diagnoses. Monkeypox signs, symptoms, and outcomes were compared according to HIV infection status. This activity was reviewed by CDC and was conducted consistent with applicable federal law and CDC policy.^¶¶^

Among 1,969 persons aged ≥18 years with monkeypox diagnosed during May 17–July 22, 2022, in eight participating jurisdictions, 755 (38%) had received an HIV diagnosis, 816 (41%) had another reportable STI diagnosed in the preceding year, and 363 (18%) had both; 1,208 (61%) persons had either (Table 1) (Table 2).*** Since May 1, 2022, 19 (1%) persons with monkeypox had received an HIV diagnosis, and 297 (15%) had received an STI diagnosis. Persons with monkeypox and HIV infection more commonly had received an STI diagnosis in the preceding year (48%) than had those without HIV infection (37%).

**TABLE 1:** Demographic characteristics of persons with monkeypox and HIV infection* — eight U.S. jurisdictions,^†^ May 17–July 22, 2022

Characteristic	No. of persons with monkeypox	No. of persons with monkeypox and diagnosed HIV infection	HIV prevalence among persons with monkeypox (row %)
**Total**	**1,969**	**755**	**38**
**Age, median, yrs (IQR)**	35 (30–42)	38 (32–45)	—
**Age group, yrs**			
18–24	106	22	21
25–34	801	246	31
35–44	670	291	43
45–54	278	131	47
≥55	105	62	59
Missing	9	3	33
**Sex assigned at birth**			
Male	1,466	548	37
Female	10	0	—
Missing or declined to answer	493	207	42
**Gender identity**			
Man	1,888	730	39
Woman	7	1	14
Transgender man or woman	8	0	—
Another gender identity	14	2	14
Missing or declined to answer	52	22	42
**Race and ethnicity**			
Asian, non-Hispanic	89	20	22
Black or African American, non-Hispanic	409	256	63
Hispanic or Latino^§^	158	64	41
Other^¶^	169	61	36
White, non-Hispanic	919	255	28
Missing	225	99	44
Monkeypox report date**			
May 15–Jun 4	24	3	13
Jun 5–11	35	9	26
Jun 12–18	64	13	20
Jun 19–25	110	32	29
Jun 26–Jul 2	201	65	32
July 3–9	331	104	31
Jul 10–16	498	196	39
Jul 17–23	596	264	44
Missing	110	69	63

* Persons with self-reported HIV infection who did not match to local HIV surveillance data (39) were excluded from the analysis.

^†^ Eight state and city or county jurisdictions independently funded for HIV surveillance: California (including Los Angeles County and San Francisco), District of Columbia, Georgia, Illinois (including Chicago), and New York (excluding New York City).

^§^ Hispanic or Latino persons can be of any race.

^¶^ Other includes persons who identify as Native Hawaiian and other Pacific Islander, American Indian or Alaska Native, or multiracial, and persons who declined to report.

** Report date includes either date of specimen collection, *Orthopoxvirus* test, monkeypox diagnosis by clinician, illness onset, or rash onset. Report date shown by epidemiologic week; the first 3 weeks of the outbreak are combined because of small numbers.

**TABLE 2:** Monkeypox hospitalization, sexually transmitted infections, and HIV prevention and care characteristics, by HIV infection status* — eight U.S. jurisdictions,^†^ May 17–July 22, 2022

Characteristic	No. (%) of persons with monkeypox^§^	No. (%) of persons without diagnosed HIV infection^§^	No. (%) of persons with diagnosed HIV infection^§^
**Total**	**1,969**	**1,214**	**755**
**Hospitalization during monkeypox illness**			
Hospitalized for monkeypox^¶^	68 (5)	26 (3)	42 (8)
Duration of hospitalization, median, days (range)**	3 (0–10)	3 (0–10)	2 (0–7)
**History of STIs**			
Reportable STI diagnosis during preceding yr	816 (41)	453 (37)	363 (48)
Gonorrhea	546 (28)	307 (25)	239 (32)
Chlamydia	489 (25)	278 (23)	211 (28)
Syphilis	165 (8)	69 (6)	96 (13)
STI diagnosis since May 1, 2022	297 (15)	166 (14)	131 (17)
**No. of STIs diagnosed during preceding yr**			
1	396 (20)	220 (18)	176 (23)
2	222 (11)	117 (10)	105 (14)
≥3	198 (10)	116 (10)	82 (11)
**HIV prevention and care characteristic**			
Received HIV care in preceding yr^††^	NA	NA	713 (94)
Suppressed HIV viral load^§§^	NA	NA	618 (82)
Recent CD4 count cells/*μ*L, median (IQR)^¶¶^	NA	NA	639 (452–831)
CD4 count <350 cells/*μ*L	NA	NA	91 (12)
CD4 count <200 cells/*μ*L	NA	NA	25 (3)
Yrs since HIV diagnosis, median (IQR)	NA	NA	10 (6–15)
HIV diagnosis since May 1, 2022	NA	NA	19 (3)
Current HIV PrEP use***	NA	115 (67)	NA

**Abbreviations:** NA = not applicable; PrEP = preexposure prophylaxis; STI = sexually transmitted infection.

* Persons with self-reported HIV infection who did not match to local HIV surveillance data (39) were excluded from the analysis.

^†^ Eight state and city or county jurisdictions independently funded for HIV surveillance: California (including Los Angeles County and San Francisco), District of Columbia, Georgia, Illinois (including Chicago), and New York (excluding New York City).

^§^ Row percentages calculated using nonmissing data.

^¶^ Overall, 1,308 persons had data available for hospitalization, including 798 persons without diagnosed HIV infection and 510 persons with diagnosed HIV infection.

** Overall, 48 hospitalized persons had data available for hospitalization duration, including 18 persons without diagnosed HIV infection and 30 persons with diagnosed HIV infection.

^††^ Receipt of HIV care was defined as at least one HIV viral load or CD4 test since May 1, 2021; tests conducted during evaluation for monkeypox might have been included.

^§§^ HIV viral suppression was defined as the most recent HIV viral load <200 copies/mL since May 1, 2021.

^¶¶^ Recent CD4 count was defined as the most recent CD4 count since May 1, 2021.

*** Among persons without diagnosed HIV infection, 172 persons had data available for current HIV PrEP use.

Among persons with monkeypox, the weekly percentage with concurrent HIV infection increased over time (31%–44% by July). The percentage of persons with monkeypox who had HIV infection was higher in older age groups: among persons aged 18–24 years, HIV prevalence was 21%, and among those aged ≥55 years, was 59%. HIV prevalence among persons with monkeypox also varied by race and ethnicity, ranging from a high of 63% among non-Hispanic Black or African American (Black) persons, to 41% among Hispanic or Latino (Hispanic) persons, 28% among non-Hispanic White persons, and 22% among non-Hispanic Asian persons.

Among 755 persons with monkeypox and HIV infection, 713 (94%) received HIV care in the preceding year, 618 (82%) were virally suppressed, and 586 (78%) had CD4 count ≥350/*μ*L. The median interval since HIV diagnosis was 10 years (IQR = 6–15 years). Data on HIV PrEP use were available for 172 (14%) persons without HIV infection, 115 (67%) of whom reported current PrEP use.

Compared with persons with monkeypox who did not have HIV infection, those with HIV infection were more likely to report rectal pain (34% versus 26%), tenesmus (20% versus 12%), rectal bleeding (19% versus 12%), purulent or bloody stools (15% versus 8%), and proctitis (13% versus 7%), but were less likely to report lymphadenopathy (48% versus 53%) (Figure). The prevalence of other signs and symptoms was similar among persons with monkeypox with and without HIV infection. Among 564 persons with monkeypox, HIV, known HIV viral load values, and signs and symptoms data, the 51 persons with unsuppressed HIV viral load were more likely than were the 513 with suppressed viral load to have lymphadenopathy (59% versus 46%), generalized pruritis (59% versus 42%), rectal bleeding (25% versus 18%), and purulent or bloody stools (22% versus 14%). Compared with persons with CD4 counts ≥350/*μ*L, those with CD4 counts <350/*μ*L more commonly experienced fever (69% versus 59%) and generalized pruritis (53% versus 42%).

**FIGURE:**
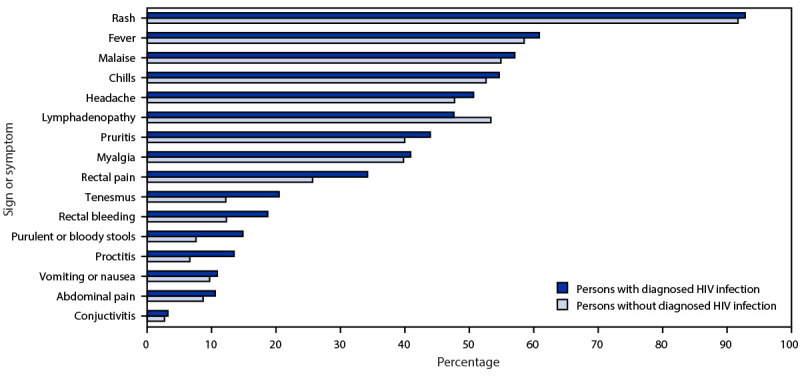
Signs and symptoms of monkeypox,*^,^^†^ by HIV infection status^§^ — eight U.S. jurisdictions,^¶^ May 17–July 22, 2022 * Persons with self-reported HIV infection who did not match to local HIV surveillance data (39) were excluded from the analysis. ^†^ Signs and symptoms were not mutually exclusive. ^§^ Percentages calculated using nonmissing data. Overall, 1,707 persons had data available for signs and symptoms except proctitis, including 1,082 persons without diagnosed HIV infection and 625 persons with diagnosed HIV infection. For proctitis, data were available for 393 persons without diagnosed HIV infection and 304 persons with diagnosed HIV infection. ^¶^ Eight state and city or county jurisdictions independently funded for HIV surveillance: California (including Los Angeles County and San Francisco), District of Columbia, Georgia, Illinois (including Chicago), and New York (excluding New York City).

Among 1,308 (66%) persons with information on hospitalization, the proportion of persons hospitalized with monkeypox was lower among those without HIV infection (3%, 26 of 798) than among those with HIV infection (8%, 42 of 510). Among 45 persons with monkeypox and HIV infection who were not virally suppressed, 12 (27%) were hospitalized, and among 61 with a CD4 count <350 cells/*μ*L, nine (15%) were hospitalized.

## Discussion

Among persons with monkeypox in eight U.S. jurisdictions, prevalences of concurrent HIV infection and reportable STI diagnoses within the preceding 12 months were high, consistent with previous reports (*1*–*5*). To date, most U.S. monkeypox cases have occurred among MSM (*4*), who have higher prevalences of HIV infection and STIs than the general population. However, in this analysis, the percentage of persons with monkeypox who had HIV infection (38%) was higher than national HIV prevalence estimates for U.S. MSM (23%); this finding was also true when comparing *Monkeypox virus* and HIV coinfection among Black persons (63%), Hispanic persons (41%), and persons aged ≥55 years (59%) to overall HIV prevalences among Black MSM (39%), Hispanic MSM (19%), and MSM aged 50–60 years (32%), respectively (*9*). Increasing HIV prevalence among persons with monkeypox over time suggests that monkeypox might be increasingly transmitted among networks of persons with HIV infection, underscoring the importance of leveraging HIV and STI care and prevention delivery systems for monkeypox vaccination and prevention efforts.^†††^ Consideration should be given to prioritizing persons with HIV infection and STIs for vaccination and other prevention efforts. HIV and STI screening and other recommended preventive care^§§§^ should be routinely offered to persons evaluated for monkeypox, with linkage to HIV care or HIV PrEP, as appropriate.

The proportion of persons with *Monkeypox virus *and HIV coinfection who received HIV care (94%) exceeded the overall percentage of persons with diagnosed HIV infection who received care in 2020 (74%) (*8*). Approximately two thirds of persons with monkeypox without HIV infection for whom data were available reported HIV PrEP use, whereas nationally, an estimated 25% of eligible persons received an HIV PrEP prescription in 2020 (*8*). Moreover, 41% of persons with monkeypox had received a diagnosis of another reportable STI in the preceding year. These findings suggest that reported monkeypox cases are occurring among persons with recent access to HIV and sexual health services. Referral bias might partially explain these findings, as persons with monkeypox signs and symptoms who have established connections with HIV or sexual health providers might be more likely to seek care (*2*), and these providers might be more likely to recognize and test for *Monkeypox virus*. Monkeypox signs and symptoms might have led persons with HIV infection who have not been in HIV care to reengage in care. Persons with monkeypox signs and symptoms who are not engaged in routine HIV or sexual health care, or who experience milder signs and symptoms, might be less likely to have their *Monkeypox virus* infection diagnosed. To ensure appropriate diagnosis and treatment, it is important that health care providers who do not specialize in HIV or sexual health become familiar with the clinical guidance for monkeypox diagnosis and treatment.^¶¶¶^

The higher prevalence of rectal signs and symptoms among persons with HIV infection could be related to differences in site of exposure, increased biologic susceptibility, or other factors. Rectal signs and symptoms did not vary by HIV immune status (CD4 count <350/*μ*L versus ≥350 *μ*L), supporting differences in site of exposure as a likely explanation. In a prospective cohort in Spain, MSM with monkeypox who engaged in receptive anal sex were more likely to report proctitis and systemic signs and symptoms preceding rash (*3*). When evaluating patients with rectal signs and symptoms, care providers should consider monkeypox and the possibility of concurrent rectal STIs. Understanding whether rectal signs and symptoms can precede rash onset or occur when rash is absent or unrecognized (because of anatomic site or small number of lesions) will help inform guidance for *Monkeypox virus* testing and new diagnostic approaches.

Limited data suggest that persons with HIV infection, particularly those with low CD4 counts or without HIV viral suppression, were more commonly hospitalized during their monkeypox illness than were persons without HIV infection. However, because data on reason for hospitalization are incomplete, it is not known whether this represents more severe monkeypox illness. Ongoing monitoring of outcomes of monkeypox by HIV infection status is important (*7*).

The findings in this report are subject to at least five limitations. First, this analysis was limited to diagnosed and reported monkeypox cases in eight jurisdictions and might not be generalizable to all U.S. monkeypox cases. Second, incomplete data on clinical signs and symptoms and hospitalization might affect the associations observed by HIV infection status. Third, some persons with undiagnosed HIV infection might have been misclassified as not having HIV, which could reduce differences in outcomes by HIV infection status. Fourth, local matching might have underestimated the prevalences of HIV infection and STIs by not including diagnoses reported in other jurisdictions or recent diagnoses. Finally, this analysis did not assess the relative contribution of structural, social, behavioral, or biologic factors to higher HIV infection and STI prevalences among persons with monkeypox. Further studies could improve understanding of such factors, monkeypox outcomes, and the impact of vaccination and treatment.

Public health efforts should continue to ensure equitable access to monkeypox screening, prevention, and treatment, particularly among MSM. It is important that systems for delivering HIV and STI care and prevention be leveraged for monkeypox evaluation, vaccination and other prevention interventions, and treatment (*10*). Data on diagnosis of HIV infections and STIs in close temporal association to monkeypox diagnosis reinforce the importance of offering recommended testing, prevention, and treatment services for HIV, STIs, and other syndemic conditions to MSM and other persons evaluated for monkeypox.**** Routine matching of monkeypox, HIV, and STI surveillance data to monitor trends and clinical characteristics of persons with coinfections can further inform public health interventions.

**Summary**

**What is already known about this topic?**

In the current global monkeypox outbreak, HIV infection and sexually transmitted infections (STIs) are highly prevalent among persons with monkeypox.

**What is added by this report?**

Among 1,969 persons with monkeypox in eight U.S. jurisdictions, 38% had HIV infection, and 41% had an STI in the preceding year. Among persons with monkeypox, hospitalization was more common among persons with HIV infection than persons without HIV infection.

**What are the implications for public health practice?**

It is important to leverage systems for delivering HIV and STI care and prevention and prioritize persons with HIV infection and STIs for vaccination. Screening for HIV and other STIs and other preventive care should be considered for persons evaluated for monkeypox, with HIV care and HIV preexposure prophylaxis offered to eligible persons.

## Footnotes

* Eight state and city or county jurisdictions independently funded for HIV surveillance: California (including Los Angeles County and San Francisco), District of Columbia, Georgia, Illinois (including Chicago), and New York (excluding New York City).

†
https://www.cdc.gov/poxvirus/monkeypox/clinicians/case-definition.html

§ Soundex is a phonetic algorithm for indexing names by sound. https://www.archives.gov/research/census/soundex

¶ Persons with self-reported HIV infection whose records were not located in local HIV surveillance data were excluded from all analyses.

** Receipt of HIV care was defined as at least one HIV viral load or CD4 test since May 1, 2021; tests conducted during evaluation for monkeypox might have been included.

†† HIV viral suppression was defined as the most recent HIV viral load <200 copies/mL since May 1, 2021.

§§ Recent CD4 count was defined as the most recent CD4 count since May 1, 2021.

¶¶ 45 C.F.R. part 46.102(l)(2), 21 C.F.R. part 56; 42 U.S.C. Sect. 241(d); 5 U.S.C. Sect. 552a; 44 U.S.C. Sect. 3501 et seq.

*** Thirty-nine persons had a self-reported HIV diagnosis in monkeypox surveillance records that could not be confirmed with local HIV surveillance data and were thus excluded from analyses.

†††
https://www.cdc.gov/poxvirus/monkeypox/interim-considerations/overview.html

§§§
https://www.cdc.gov/std/treatment-guidelines/default.htm

¶¶¶
https://www.cdc.gov/poxvirus/monkeypox/clinicians/treatment.html

****
https://www.cdc.gov/msmhealth/index.htm
